# Antimicrobial Resistance Patterns and Antibiotic Use during Hospital Conversion in the COVID-19 Pandemic

**DOI:** 10.3390/antibiotics10020182

**Published:** 2021-02-11

**Authors:** Bernardo A. Martinez-Guerra, Maria F. Gonzalez-Lara, Nereyda A. de-Leon-Cividanes, Karla M. Tamez-Torres, Carla M. Roman-Montes, Sandra Rajme-Lopez, G. Ivonne Villalobos-Zapata, Norma I. Lopez-Garcia, Areli Martínez-Gamboa, Jose Sifuentes-Osornio, Edgar Ortiz-Brizuela, Eric Ochoa-Hein, Arturo Galindo-Fraga, Miriam Bobadilla-del-Valle, Alfredo Ponce-de-León

**Affiliations:** 1 Infectious Diseases Department, Instituto Nacional de Ciencias Médicas y Nutrición Salvador Zubirán, 14080 Mexico City, Mexico; bernardo.martinezg@incmnsz.mx (B.A.M.-G.); nereyda.deleon@infecto.mx (N.A.d.-L.-C.); carla.romanm@incmnsz.mx (C.M.R.-M.); sandra.rajmel@incmnsz.mx (S.R.-L.); edgar.ortizb@incmnsz.mx (E.O.-B.); 2 Clinical Microbiology Laboratory, Infectious Diseases Department, Instituto Nacional de Ciencias Médicas y Nutrición Salvador Zubirán, 14080 Mexico City, Mexico; fernanda.gonzalezl@incmnsz.mx (M.F.G.-L.); karla.tamezt@incmnsz.mx (K.M.T.-T.); yvonne.villalobosz@incmnsz.mx (G.I.V.-Z.); irene.lopezg@incmnsz.mx (N.I.L.-G.); rosa.martinezg@incmnsz.mx (A.M.-G.); miriam.bobadillav@incmnsz.mx (M.B.-d.-V.); 3 Department of Medicine, Instituto Nacional de Ciencias Médicas y Nutrición Salvador Zubirán, 14080 Mexico City, Mexico; jose.sifuenteso@incmnsz.mx; 4 Infection Control and Hospital Epidemiology Department, Instituto Nacional de Ciencias Médicas y Nutrición Salvador Zubirán, 14080 Mexico City, Mexico; eric.ochoah@incmnsz.mx (E.O.-H.); arturo.galindof@incmnsz.mx (A.G.-F.)

**Keywords:** antimicrobial use, COVID-19, hospital-acquired infections

## Abstract

Objective: To describe empirical antimicrobial prescription on admission in patients with severe COVID-19, the prevalence of Hospital-Acquired Infections, and the susceptibility patterns of the causing organisms. Methods: In this prospective cohort study in a tertiary care center in Mexico City, we included consecutive patients admitted with severe COVID-19 between March 20th and June 10th and evaluated empirical antimicrobial prescription and the occurrence of HAI. Results: 794 patients with severe COVID-19 were admitted during the study period. Empiric antibiotic treatment was started in 92% of patients (731/794); the most frequent regimes were amoxicillin-clavulanate plus atypical coverage in 341 (46.6%) and ceftriaxone plus atypical coverage in 213 (29.1%). We identified 110 HAI episodes in 74/656 patients (11.3%). Ventilator-associated pneumonia (VAP) was the most frequent HAI, in 56/110 (50.9%), followed by bloodstream infections (BSI), in 32/110 (29.1%). The most frequent cause of VAP were *Enterobacteriaceae* in 48/69 (69.6%), followed by non-fermenter gram-negative bacilli in 18/69 (26.1%). The most frequent cause of BSI was coagulase negative staphylococci, in 14/35 (40.0%), followed by *Enterobacter complex* in 7/35 (20%). Death occurred in 30/74 (40.5%) patients with one or more HAI episodes and in 193/584 (33.0%) patients without any HAI episode (*p* < 0.05). Conclusion: A high frequency of empiric antibiotic treatment in patients admitted with COVID-19 was seen. VAP and BSI were the most frequent hospital-acquired infections, due to *Enterobacteriaceae* and coagulase negative staphylococci, respectively.

## 1. Introduction

Bacterial coinfections are a major cause of morbidity and mortality among patients with viral infections of the respiratory tract such as severe influenza, with reported rates ranging from 2% to 65%. Most commonly, bacteria such as *Streptococcus pneumoniae* and *Staphylococcus aureus* account for 35% and 28% of infections, respectively [1,2].

The distinction of bacterial from viral infections is a clinical challenge that often leads to unnecessary antibiotic use [3,4]. During the SARS-CoV-2 pandemic, a multifactorial increase in antimicrobial prescriptions has been noted in patients presenting with severe COVID-19, despite a low (3.5–8%) prevalence of bacterial coinfections on admission [5,6,7]. These factors include the difficulty to obtain respiratory samples, a breakdown in surveillance and antimicrobial stewardship programs and the absence of evidence-based antiviral treatments [5,7].

Although the pandemic is a challenge to antimicrobial stewardship programs, several antimicrobial stewardship recommendations include obtaining microbiological samples on admission, early discontinuation or de-escalation once bacterial infection is ruled out and enforcing the proper treatment duration [8,9].

The long-term local and global impact of the COVID-19 pandemic in antimicrobial resistance rates and antimicrobial consumption are yet to be known.

In this study we aimed to describe the pattern of antimicrobial prescription on admission in patients with severe COVID-19, the rate of hospital-acquired infections and the susceptibility patterns of the causing organisms during the first months of the pandemic.

## 2. Material and Methods

We conducted a prospective cohort study in a tertiary care center in Mexico City, converted to a COVID-19 dedicated facility. Hospital reorganization included the expansion of the Intensive Care Unit (ICU) and redistribution of nursing and medical staff. All consecutive patients admitted with polymerase chain reaction (PCR) confirmed community-acquired severe COVID-19 between March 20th and June 10th were included. A case was defined as severe by the presence of any of the following: respiratory rate ≥30 breaths per minute, SpO2 < 93%, PaO2/FiO2 ratio < 300 and involvement of 50% or more of the lung parenchyma as evidenced by a chest CT scan [10]. Data regarding clinical presentation, demographics, laboratory results, antibacterial prescription on admission and duration, development and characteristics of hospital-acquired infections and discharge status were obtained from the electronical medical record. The primary outcome was the development of a Hospital-acquired Infection (HAI). For each HAI episode, the causative microorganism and its susceptibility pattern were recorded. Secondary outcomes included death during hospitalization and length of stay. Hospital-acquired Infections were identified and defined according to the National Healthcare Safety Network (NHSN) standardized criteria [11]. Of note, dexamethasone became a standard of care on June 30th, after the preliminary results of the RECOVERY trial were published [12]. Patients who did not meet the criteria of severe COVID-19 and who had a stay of less than 24 h were excluded from the analysis. The study was reviewed and approved by the Institutional Review Board (Ref. number 3333). Written informed consent was waived because of the observational nature of the study.

### 2.1. Laboratory Procedures

SARS-CoV-2 testing was performed on nasopharyngeal swab samples. Upper respiratory samples were transported in a universal transport medium for viruses. Nucleic acid extraction was performed using the NucliSens easyMAG system (bioMérieux, Boxtel, The Netherlands). A real-time reverse transcription-polymerase chain reaction was processed on Applied Biosystems 7500 thermocycler (Foster City, CA, USA) using primers and conditions described elsewhere [13].

### 2.2. Bacterial Isolate Identification

Clinical samples were taken during routine clinical care and sent to the Clinical Microbiology Laboratory. Bacterial isolates identification was done through Bruker (MALDI-TOF, Billerica, MA, USA) Microflex LT, using Bruker Biotyper software. The analysis used the default parameter settings. Scores of ≥2.0 were considered of high-confidence for species identifications. When the MALDI-Biotyper did not identify the isolate, we used Vitek2 system (bioMérieux, Durham, NC, USA) cards for Gram-positive and Gram-negative organisms, following the manufacturer’s instructions.

### 2.3. Susceptibility Testing

After 24 h incubation in McConkey or blood agar, bacterial colonies were retrieved and reconstituted into a 0.5 McFarland suspension (in 0.45% NaCl), and then inoculated in VITEK^®^ AST-ST03, AST-N271 and AST-N272 (bioMérieux, Durham, NC, USA) cards. Susceptibility testing was obtained using the VITEK^®^ 2 System (bioMérieux) following the manufacturer’s instructions.

For study purposes, an AmpC producer was considered in *Enterobacteriaceae* species with known chromomosomal AmpC Beta-Lactamases [14]. Isolates with Extended spectrum Beta-Lactamases (ESBL) were considered in those *Enterobacteriaceae* resistant to 3rd generation cephalosporins and monobactams [15]. Carbapenem-resistant *Enterobacteriaceae* were considered when isolates with any resistance to carbapenems in VITEK^®^ AST was confirmed through a modified CIM test [16]. Multidrug-resistant (MDR) *Pseudomonas aeruginosa* was considered in isolates with resistance to at least one agent in three or more antibiotic categories [17].

### 2.4. Statistical Analysis

Categorical variables were reported as frequencies and proportions. Quantitative variables were reported using mean and standard deviation, or median and interquartile range (IQR) according to their observed distribution. To evaluate significant statistical differences, χ^2^ was used for comparisons between proportions.

## 3. Results

During the study period, 794 patients with severe COVID-19 were admitted. Sixty-one percent (489/794) were male, the median age was 52 years (IQR 43–62), the median body mass index (BMI) was 29.7 kg/m^2^ and obesity was seen in 46.1% of patients (364/794). Upon admission, preexisting type 2 Diabetes Mellitus (T2DM) and hypertension were present in 27.2% (216/794) and 31.9% (253/794), respectively. Median time from symptom onset to admission was 8 days (IQR 6–10). On admission, patients had a median pulse oximeter oxygen saturation (SpO2) of 83% (IQR 70–88%), median leukocyte count of 8.3 × 10^3^ cells/uL (IQR 6–11.5), median procalcitonin concentration of 0.31 ng/mL (IQR 0.11–0.82) and median C-reactive protein concentration (CRP) of 157 mg/L (IQR 90–238). Multilobe lung involvement on chest CT was seen in 99% (785/794).

Demographic, clinical COVID-19 characteristics, and initial antimicrobial treatment are described in Table 1. Empiric antibiotic treatment was started in 92% (731/794); the most frequent regimes were amoxicillin-clavulanate plus atypical coverage in 341 patients (46.6%) and ceftriaxone plus atypical coverage in 213 patients (29.1%). Atypical coverage was considered when azithromycin or clarithromycin were prescribed. Broad-spectrum antimicrobials were used in 95 patients (12.9%) and COVID-19 directed therapy was indicated in 45.1% of cases (358/794). Steroids were used in 9.2% of patients. Even though diagnostic tests were not routinely performed, seven patients presented respiratory viral co-infections (three adenovirus, two respiratory syncytial virus, one rhinovirus, one influenza B) upon admission, evidenced by results of multiplex PCR in respiratory samples.

### 3.1. Hospital-Acquired Infections

In 656 patients with complete follow up, we identified 110 HAI episodes in 74 patients (11.3%). Of the 74 patients who presented HAI, 45 had one episode, 23 had two, five had three and one had four HAI episodes. The median hospital stay at the time of HAI diagnosis was 9 days (IQR 7–15 days).

Of 110 documented HAI episodes, ventilator-associated pneumonia/hospital-acquired pneumonia (VAP/HAP) was the most frequent in 56/110 (50.9%), followed by bloodstream infections (BSI) in 32/110 (29.1%), filamentous fungal infection in 14/110 (12.7%), candidemia in 6/110 (5.5%) and urinary tract infection in 2/110 (1.8%). Findings are summarized in Table 2.

Microbial isolates for VAP/HAP and BSI are summarized in Table 3. The most frequent cause of VAP/HAP were *Enterobacteriaceae* in 48/69 patients (69.6%), followed by non-fermenter gram-negative bacilli in 18/69 (26.1%). Nine episodes of VAP/HAP (16.1%) were polymicrobial. The most frequent cause of BSI was coagulase negative staphylococci in 14/35 patients (40.0%), followed by *Enterobacter complex* in 7/35 (20.0%). *Candida parapsilosis* was the most frequent agent-involved candidemia (4/6), while *Aspergillus* section Flavi was the most frequent isolate in filamentous fungal infection (5/14). Antimicrobial susceptibilities are described in Table 4.

### 3.2. Outcome

Out of 656 patients who had complete follow-up, 223 (34.0%) died during hospitalization. Death occurred in 30/74 (40.5%) patients with one or more HAI episodes and in 193/584 (33.0%) patients without any HAI episode (*p* < 0.05). The median length of stay was 7 days (IQR 4–13) among the 656 patients who completed follow-up. Among the patients who died during follow-up, median length of stay until death was 6 days (IQR 3–10). Length of stay was significantly higher in patients who developed a hospital-acquired infection [median length of 27 days (IQR 18–36) vs 6 days (IQR 4–6), *p* < 0.01].

Among 794 patients admitted during the study period, 433 (54.5%) were discharged, 223 (28.1%) died, 124 (15.6%) were transferred to another hospital and 14 (1.8%) were discharged against medical advice.

## 4. Discussion

We conducted a prospective study to describe the pattern of empiric antibiotic prescriptions and HAIs in severe COVID-19 patients admitted to a tertiary center in Mexico City. We found widespread empiric antimicrobial prescriptions of Beta-lactams and atypical coverage, which is consistent with the recommended community-acquired pneumonia regime. In our study 92% of patients received empirical antibiotic treatment, which differs from Vaughn et al., who reported empiric antibiotic use in 56.6% of patients with COVID-19 admitted to 38 hospitals in Michigan [18]. In that study, empiric antibiotic use ranged from 27% to 84% among hospitals, and the most frequently prescribed antibiotic was ceftriaxone in 38.9%. Although the prevalence of bacterial coinfections upon admission is low (2.4–7%), empiric antimicrobial treatment is extremely common (72–90%) [5,7,19]. The excessive antimicrobial prescription we found may be due to the disruption of antimicrobial stewardship programs due to workforce redistribution during the pandemic and decreased surveillance during the initial months of the pandemic. Increased antimicrobial use may also be derived from the experience with patients with severe influenza, who have bacterial coinfections upon admission (11–35%) [6] more commonly. Likewise, the high frequency of atypical coverage we observed may have been due to the widespread use of azithromycin during the early phase of the pandemic in our country and worldwide. Because of the low prevalence of bacterial and atypical coinfections, the WHO has advised against routine empirical antibiotic treatment in patients without severe COVID-19 and against routine atypical coverage [19]. In this study, increased disease severity upon admission may explain the widespread use of antimicrobial agents. Despite these recommendations, increasing antibiotic use, particularly ceftriaxone and azithromycin, has been reported. Unfortunately, we were unable to recover the true incidence of atypical infections, since we did not routinely perform such diagnostic tests upon admission. Viral co-infections are uncommon, and further studies are required to understand their impact on the outcomes [20]. No culture-proven community-acquired bacterial co-infections were identified during the study period.

In our study, 11.3% of the patients presented at least one episode of HAI. The observed frequency of HAI is similar to other reports. Ji et al. reported that 6.8% of patients admitted to a hospital in Wuhan, China, presented secondary bacterial infections [21]. In their review and meta-analysis, Langford et al. reported that 14.3% of patients developed a secondary bacterial infection [22]. A higher incidence of HAIs among patients with critical COVID-19 has been described [23]. Of the 110 episodes of HAI in our report, the most common source of infection (51%) was pneumonia, followed by bloodstream infections in 29%. The most frequent cause of VAP/HAP in our report was *Enterobacteriaceae,* cultured in 70% of cases. Pulmonary infections are the most frequent type of secondary infections reported, mainly in critically ill patients [22]. Li J et al. reported *A. baumannii*, *K. pneumoniae* and S. *maltophilia* as the most frequent pathogens involved in secondary lung infections in patients with COVID-19 [21]. The latter is due to high-frequency invasive procedures such as orotracheal intubation and mechanical ventilation in such patients. The high proportion of *Enterobacteriaceae* from respiratory isolates in our center reinforces the importance of antimicrobial stewardship in order to avoid the antibiotic-driven selection of highly resistant strains. It also underscores the importance of following VAP preventive bundles. The most frequent causative microorganisms of bloodstream infections were coagulase-negative Staphylococci, at 40%, which is similar to the 37.2% reported by Jie Li et al. [21]. Attention to the preventive measures in the management of vascular lines is underscored. Invasive pulmonary aspergillosis has been described in critically ill patients with COVID-19–associated acute respiratory distress syndrome. This complication should be considered in high-risk patients. Extended amoxicillin and ceftriaxone use in our center may partly explain the high frequency of AmpC and ESBL producers in our report. Rational antimicrobial use should be reinforced, and antimicrobial stewardship programs must be implemented [24]. After the study period, the antimicrobial stewardship program was reinforced. Strategies such as rational use of procalcitonin, Streptococcal Urine Antigen use, prompt de-escalation and early antimicrobial interruption are constantly encouraged during daily rounds and weekly meetings. An analysis of the effectiveness of stewardship strategies in our center is pending. Moreover, infection control policies such as VAP and BSI prevention bundles were reinforced with educational sessions and weekly meetings. Notably, the length of stay was longer in patients who developed at least one HAI episode, as previously described [25]. The latter could be explained by the high frequency of invasive mechanical ventilation among most patients that eventually developed HAI.

We acknowledge several limitations in this study. The true rate of co-infections upon admission is unknown because diagnostic tests (e.g., multiplex PCR-panel for respiratory pathogens) are not routinely performed. Also, the role of procalcitonin upon admission cannot be established since it was not systematically collected. Importantly, it must be noted that the reported data was obtained before the widespread use of dexamethasone; hence, mortality data should be interpreted with caution. Although hydroxychloroquine prescriptions were frequent during the initial months of the pandemic, its use was actively discouraged since April 2020 due to increasing evidence of futility. We do not believe that the decrease in hydroxychloroquine use had an impact in the reported outcomes, as it has been reported previously to be futile in the management of COVID-19 patients. [26,27] Additionally, our population consisted mostly of males. Obesity, T2DM and hypertension were common and are known predictors of a worse outcome [28]. A relevant finding was the high frequency of obesity in our cohort. This finding could be related to the high prevalence seen in Mexico. A prevalence of 39.6% of obesity in Mexican COVID-19 patients was initially described by Ortiz-Brizuela et al. [29]. Similar rates have been observed (36% to 62%) in patients with severe influenza [30,31]. Incomplete follow-up occurred in 17% and was mainly due to the following reasons: patients with impending need of mechanical ventilation and unavailable ICU at the moment were transferred to close hospitals with available ICU, and patients with decreasing oxygen flow support and improvement were candidates to be transferred to convalescence centers. The absence of these data might have led to selection bias and overrepresentation of cases with HAIs. A comparison of demographic, and baseline clinical and laboratory characteristics between patients that had a complete versus a non-complete follow-up is provided in the supplementary material (Table S1). 

We believe our results reflect the antibiotic prescribing practices during the early months of the pandemic and may be generalized to most hospitals reconverted into COVID-19 centers in our region. We believe that antimicrobial stewardship is a critical program that should be reinforced rather that underscored, despite heavy workloads. COVID-19 centers should consider measuring antimicrobial use, develop local guidelines and monitor antimicrobial resistance emergence while reinforcing infection control strategies.

## 5. Conclusions

A high frequency of empiric antibiotic treatment in patients admitted with severe COVID-19 was observed. Ventilator-associated pneumonia and bloodstream infections were the most frequent hospital-acquired infections, due to Enterobacteriaceae and coagulase negative staphylococci, respectively. Antimicrobial stewardship programs must be reinforced. Further studies to describe the impact of the COVID-19 pandemic in antimicrobial resistance are needed.

## Figures and Tables

**Table 1:** Demographic, clinical, baseline laboratory and initial treatment in patients with severe COVID-19.

Characteristic	*N* = 794 (100%)
Male sex, *n* (%)	489 (61.6)
Age years, median (IQR)	52 (43–62)
BMI kg/m^2^, median (IQR) (*n* = 755)	29.7 (26.7–33.2)
Obesity, *n* (%) (*n* = 790)	364 (46.1)
Overweight, *n* (%) (*n* = 779)	295 (37.9)
Type 2 diabetes mellitus, *n* (%)	216 (27.2)
Immunosuppression, *n* (%)	45 (5.7)
Hypertension, *n* (%)	253 (31.9)
HIV infection, *n* (%)	10 (1.3)
Ischemic heart disease, *n* % (*n* = 461)	19 (4.2)
Chronic kidney disease, *n* (%)	24 (3.0)
Liver failure, *n* (%) (*n* = 790)	5 (0.63)
Smoking history, *n* (%) (*n* = 785)	117 (14.9)
Charlson score ≥ 2, *n* (%)	328 (41.4)
Multilobe involvement in CT, *n* (%) (*n* = 793)	785 (99)
Arterial blood oxygen saturation, median (IQR) (*n* = 770)	83 (70–88)
Days since symptom onset, median (IQR)	8 (6–10)
Total white blood cell count ×10^3^/uL, median (IQR) (*n* = 789)	8.3 (6.0–11.5)
Procalcitonin ng/mL, median (IQR) (*n* = 140)	0.31 (0.11–0.82)
C-reactive protein mg/L median (IQR) (*n* = 766)	157.1 (90.0–238.8)
Empiric antibiotic treatment, *n* (%)	731 (92)
Antibiotic treatment, *n* (%)	731 (91.9)
Amoxicillin + atypical coverage	341 (46.6)
Ceftriaxone + atypical coverage	213 (29.1)
Ceftriaxone or amoxicillin monotherapy	35 (4.7)
Atypical coverage monotherapy	54 (7.4)
Broad specter Beta-lactam + atypical	52 (7.1)
Broad specter Beta-lactam + vancomycin	18 (2.4)
Broad specter Beta-lactam monotherapy	25 (3.4)
Other	2 (0.3)
COVID-19-directed therapy, *n* (%)	358 (45.1)
Hydroxychloroquine/chloroquine, *n* (%)	219 (27.6)
Steroid use, *n* (%)	73 (9.2)
ICU admission, *n* (%)	205 (25.8)
Mechanical ventilation, *n* (%)	188 (23.7)
Days on mechanical ventilation, median (IQR)	12 (7–17)

ICU admission at any point during stay was required in 205/794 patients (25.8%) and invasive mechanical ventilation in 188/794 (23.7%). The median duration of invasive mechanical ventilation was 12 days (IQR 7–17 days).

**Table 2:** Hospital-acquired infections among patients with COVID-19 who completed follow up.

Characteristic	*n* = 656
Number of patients with at least one HAI episode	74 (11.3)
Ventilator o healthcare-associated pneumonia	49 (7.5)
Bloodstream infection	27 (4.1)
Invasive mold infection	14 (2.1)
Candidemia	6 (0.9)
Urinary tract infection	2 (0.3)
Number of different HAI episodes	110 (100)
Ventilator o healthcare-associated pneumonia	56 (50.9)
Bloodstream infection	32 (29.1)
Invasive mold infection	14 (12.7)
Candidemia	6 (5.5)
Urinary tract infection	2 (1.8)

**Table 3:** Microbial isolates in ventilator-associated pneumonia/hospital-acquired pneumonia and bloodstream infections.

Microbial isolates in 56 episodes of VAP/HAP	69 (100)
*Enterobacter complex*	29 (42.0)
*Pseudomonas aeruginosa*	10 (14.5)
*Klebsiella spp*	9 (13.0)
*Escherichia coli*	9 (13.0)
*Stenotrophomonas maltophilia*	6 (8.7)
Other	6 (8.7)
Microbial isolates in 32 episodes of BSI	35 (100)
*Coagulase negative staphylococci*	14 (40.0)
*Enterobacter complex*	7 (20.0)
*Enterococcus spp*	6 (17.1)
*Pseudomonas aeruginosa*	3 (8.6)
Other	5 (14.3)

**Table 4:** Antimicrobial susceptibility patterns in HAI episodes during the COVID-19 pandemic.

	Total *n* = 127	VAP/HAP *n* = 69	BSI *n* = 35
AmpC producers	37 (29.1)	26 (37.7)	5 (14.3)
ESBL producers	7 (5.5)	6 (8.7)	1 (2.9)
MDR P. Aeruginosa	1 (0.8)	-	1 (2.9)
CRE	4 (3.1)	3 (4.3)	0
Azole resistant *Candida*	5/6 (83.3)	-	5/6 (83.3)

ESBL: Extended spectrum beta-lactamase. MDR: Multidrug resistant. CRE: Carbapenem-resistant Enterobacteriaceae.

## Data Availability

The data presented in this study are available on request from the corresponding author. The data are not publicly available due to authors preferences.

## Supplementary Materials

The Table S1 available online at https://www.mdpi.com/2079-6382/10/2/182/s1.

## References

[1] Klein E.Y., Monteforte B., Gupta A., Jiang W., May L., Hsieh Y., Dugas A. (2016). The frequency of influenza and bacterial coinfection: A systematic review and meta-analysis. Influenza Other Respi. Viruses.

[2] Huttner B.D., Catho G., Pano-Pardo J.R., Pulcini C., Schouten J. (2020). COVID-19: Don’t neglect antimicrobial stewardship principles!. Clin. Microbiol. Infect..

[3] Gonzales R., Bartlett J.G., Besser R.E., Cooper R.J., Hickner J.M., Hoffman J.R., Sande M.A. (2001). Principles of appropriate antibiotic use for treatment of acute respiratory tract infections in adults: Background, specific aims, and methods. Ann. Emerg. Med..

[4] Metlay J.P., Camargo C.A., MacKenzie T., McCulloch C., Maselli J., Levin S.K., Kersey A., Gonzales R. (2007). Cluster-Randomized Trial to Improve Antibiotic Use for Adults With Acute Respiratory Infections Treated in Emergency Departments. Ann. Emerg. Med..

[5] Abelenda-Alonso G., Padullés A., Rombauts A., Gudiol C., Pujol M., Alvarez-Pouso C., Jodar R., Carratalà J. (2020). Antibiotic prescription during the COVID-19 pandemic: A biphasic pattern. Infect. Control. Hosp. Epidemiol..

[6] Nestler M., Godbout E., Lee K., Kim J., Noda A.J., Taylor P., Pryor R., Markley J.D., Doll M., Bearman G. (2020). Impact of COVID-19 on pneumonia-focused antibiotic use at an academic medical center. Infect. Control. Hosp. Epidemiol..

[7] Rawson T.M., Moore L.S.P., Castro-Sanchez E., Charani E., Davies F., Satta G., Ellington M.J., Holmes A.H. (2020). COVID-19 and the potential long-term impact on antimicrobial resistance. J. Antimicrob. Chemother..

[8] Furukawa D., Graber C.J. (2020). Antimicrobial Stewardship in a Pandemic: Picking Up the Pieces. Clin. Infect. Dis..

[9] Martin E., Philbin M., Hughes G., Bergin C., Talento A.F. (2021). Antimicrobial stewardship challenges and innovative initiatives in the acute hospital setting during the COVID-19 pandemic. J. Antimicrob. Chemother..

[10] Wu Z., McGoogan J.M. (2020). Characteristics of and Important Lessons From the Coronavirus Disease 2019 (COVID-19) Outbreak in China: Summary of a Report of 72 314 Cases From the Chinese Center for Disease Control and Prevention. JAMA.

[11] CDC, NCEZID, DHQP National Healthcare Safety Network (NHSN) Patient Safety Component Manual. https://www.cdc.gov/ncezid/dhqp/index.html.

[12] Horby P., Lim W.S., Emberson J.R., Mafham M., Bell J.L., Linsell L., Staplin N., Brightling C., Ustianowski A., The RECOVERY Collaborative Group (2020). Dexamethasone in Hospitalized Patients with Covid-19—Preliminary Report. N. Engl. J. Med..

[13] Corman V.M., Landt O., Kaiser M., Molenkamp R., Meijer A., Chu D.K., Bleicker T., Brünink S., Schneider J., Schmidt M.L. (2020). Detection of 2019 -nCoV by RT-PCR. Eur. Surveill..

[14] Jacoby G.A. (2009). AmpC Β-Lactamases. Clin. Microbiol. Rev..

[15] Ghafourian S., Sadeghifard N., Soheili S., Sekawi Z. (2015). Extended Spectrum Beta-lactamases: Definition, Classification and Epidemiology. Curr. Issues Mol. Biol..

[16] Carbapenemase Producing Carbapenem-Resistant Enterobacteriaceae (CP-CRE) 2018 Case Definition. https://wwwn.cdc.gov/nndss/conditions/carbapenemase-producing-carbapenem-resistant-enterobacteriaceae/case-definition/2018/.

[17] Horcajada J.P., Montero M., Oliver A., Sorlí L., Luque S., Gómez-Zorrilla S., Benito N., Grau S. (2019). Epidemiology and treatment of multidrug-resistant and extensively drug-resistant Pseudomonas aeruginosa infections. Clin. Microbiol. Rev..

[18] Vaughn V.M., Gandhi T., Petty L.A., Patel P.K., Prescott H.C., Malani A.N., Ratz D., McLaughlin E., Chopra V., A Flanders S. (2020). Empiric Antibacterial Therapy and Community-Onset Bacterial Co-Infection In Patients Hospitalized with COVID-19: A Multi-Hospital Cohort Study. Clin. Infect. Dis..

[19] Lansbury L., Lim B., Baskaran V., Lim W.S. (2020). Co-infections in people with COVID-19: A systematic review and meta-analysis. J. Infect..

[20] World Health Organization (2020). Clinical Management of COVID-19: Interim Guidance.

[21] Nowak M.D., Sordillo E.M., Gitman M.R., Paniz Mondolfi A.E. (2020). Coinfection in SARS-CoV-2 infected patients: Where are influenza virus and rhinovirus/enterovirus?. J. Med. Virol..

[22] Li J., Wang J., Yang Y., Cai P., Cao J., Cai X., Zhang Y. (2020). Etiology and antimicrobial resistance of secondary bacterial infections in patients hospitalized with COVID-19 in Wuhan, China: A retrospective analysis. Antimicrob. Resist Infect. Control.

[23] Langford B.J., So M., Raybardhan S., Leung V., Westwood D., MacFadden D.R., Soucy J.R., Daneman N. (2020). Bacterial co-infection and secondary infection in patients with COVID-19: A living rapid review and meta-analysis. Clin. Microbiol. Infect..

[24] Sieswerda E., de Boer M.G.J., Bonten M.M.J., Boersma W.G., Jonkers R.E., Aleva R.M., Kullberg B., Schouten J.A., van de Garde E.M.W., Verheij T.J. (2020). Recommendations for antibacterial therapy in adults with COVID-19—An evidence based guideline. Clin. Microbiol. Infect..

[25] Falcone M., Tiseo G., Giordano C., Leonildi A., Menichini M., Vecchione A., Pistello M., Guarracino F., Ghiadoni L., Forfori F. (2020). Predictors of hospital-acquired bacterial and fungal superinfections in COVID-19: A prospective observational study. J. Antimicrob. Chemother..

[26] Self W.H., Semler M.W., Leither L.M., Casey J.D., Angus D.C., Brower R.G., Chang S.Y., Collins S.P., Eppensteiner J.C., Filbin M.R. (2020). Effect of Hydroxychloroquine on Clinical Status at 14 Days in Hospitalized Patients with COVID-19: A Randomized Clinical Trial. JAMA J. Am. Med. Assoc..

[27] Cavalcanti A.B., Zampieri F.G., Rosa R.G., Azevedo L.C., Veiga V.C., Avezum A., Damiani L.P., Marcadenti A., Kawano-Dourado L., Lisboa T. (2020). Hydroxychloroquine with or without Azithromycin in Mild-to-Moderate Covid-19. N. Engl. J. Med..

[28] Bello-Chavolla Y., Bahena-López J.P., Villa N.E.A., Vargas-Vázquez A., González-Díaz A., Márquez-Salinas A., Fermín-Martínez C.A., Naveja J.J., Aguilar-Salinas C.A. (2015). Predicting mortality due to SARS-CoV-2: A mechanistic score relating obesity and diabetes to COVID-19 outcomes in Mexico Omar. J. Clin. Endocrinol. Metab..

[29] Ortiz-Brizuela E., Villanueva-Reza M., González-Lara M.F., Tamez-Torres K.M., Román-Montes C.M., Díaz-Mejía B.A., Pérez-García E., Olivas-Martínez A., Rajme-López S., Martinez-Guerra B.A. (2020). Clinical and Epidemiological Characteristics of Patients Diagnosed With Covid-19 in a Tertiary Care Center in Mexico City: A Prospective Cohort Study. Rev. Invest. Clin..

[30] Domınguez-Cherit G., Lapinsky S.E., Macias A.E., Pinto R., Espinosa-Perez L., Poblano-Morales M., Baltazar-Torres J.A., Bautista E., Martinez A., de la Torre A. (2009). 2009 Influenza A (H1N1) in Mexico. JAMA.

[31] Hernández-Cárdenas C.M., Serna-Secundino H., García-Olazarán J.G., Aguilar-Pérez C.L., Rocha-Machado J., Campos-Calderón L.F., Lugo-Goytia G. (2016). Acute Respiratory Distress Syndrome Secondary to Influenza A(H1N1)pdm09: Clinical Characteristics and Mortality Predictors. Rev. Invest. Clin..

