# Supplementary material for: Antimicrobial Resistance Patterns and Antibiotic Use during Hospital Conversion in the COVID-19 Pandemic
Source: Antibiotics (Basel). 2021 Feb 11;10(2):182. doi: 10.3390/antibiotics10020182 (PMC7917840; doi:10.3390/antibiotics10020182)
Supplement: Supplementary file 1 [file antibiotics-10-00182-s001.pdf]

Table S1. Comparison of demographic, and baseline clinical and laboratory characteristics between patients that had a complete versus a non-complete follow-up

Characteristic	All patients n= 794 (100%)	Patients with complete follow-up n=656 (82.6%)	Patients with incomplete follow-up n=138 (17.4%)	p
Male sex, n (%)	489 (61.6)	397 (60.5)	92 (66.7)	0.177
Age years, median (IQR)	52 (43 - 62)	52 (43-62)	52 (42-61)	0.5063
BMI kg/m2, median (IQR) (n=755)	29.7 (26.7 - 33.2)	29.4 (26.6-33.1)	30.4 (28.0-34.6)	0.0218
Obesity, n (%) (n=790)	364 (46.1)	295 (45.1)	69 (50.7)	0.231
Overweight, n (%) (n=779)	295 (37.9)	247 (38.2)	48 (36.1)	0.642
Type 2 diabetes mellitus, n (%)	216 (27.2)	177 (27.0)	39 (28.3)	0.759
Immunosuppression, n (%)	45 (5.7)	41 (6.3)	4 (2.9)	0.156*
Hypertension, n (%)	253 (31.9)	213 (32.5)	40 (29.0)	0.425
HIV infection, n (%)	10 (1.3)	10 (1.52)	0	0.225*
Ischemic heart disease, n % (n=461)	19 (4.1)	17 (4.6)	2 (2.2)	0.391*
Chronic kidney disease, n (%)	24 (3.0)	22 (3.4)	2 (1.5)	0.408*
Liver failure, n (%) (n=790)	5 (0.63)	5 (0.8)	0	0.594*
Smoking history, n (%) (n=785)	117 (14.9)	92 (14.2)	25 (18.1)	0.243
Charlson score, median (IQR)	1 (0 - 2)	1 (0-3)	1 (0-2)	0.3991
Multilobe involvement in CT, n (%) (n=793)	785 (99)	648 (98.9)	137 (99.3)	1.0
Arterial blood oxygen saturation, median (IQR) (n=770)	83 (70 - 88)	83 (70-88)	82 (74-87)	0.6973

Days since symptom onset, median (IQR)	8 (6 - 10)	7 (6-10)	8 (6-11)	0.2845
Total white blood cell count x10^3/uL, median (IQR) (n=789)	8.3 (6.0 - 11.5)	8.1 (5.9-11.5)	8.7 (6.5-11.6)	0.1509
Procalcitonin ng/mL, median (IQR) (n=140)	0.31 (0.11 - 0.82)	0.31 (0.10-0.86)	0.30 (0.13-0.58)	0.7676
C-reactive protein mg/L median (IQR) (n=766)	157.1 (90.0 – 238.8)	151.8 (84.5-236.3)	172.5 (123.4-248.7)	0.0262

*Fisher exact test was performed.
